# Population Pharmacokinetics and Model-Informed Dose Optimization of Teicoplanin in Adults with Hematological Malignancies

**DOI:** 10.3390/pharmaceutics18010100

**Published:** 2026-01-12

**Authors:** María García-Hervalejo, José Germán Sánchez-Hernández, Irene Conde-González, Alejandro Avendaño Pita, María José Otero

**Affiliations:** 1 Pharmacy Service, University Hospital of Salamanca, 37007 Salamanca, Spain; 2 Biomedical Research Institute of Salamanca (IBSAL), 37007 Salamanca, Spain; 3 Department of Pharmaceutical Sciences, Faculty of Pharmacy, University of Salamanca, 37007 Salamanca, Spain; 4 Hematology Service, University Hospital of Salamanca, 37007 Salamanca, Spain

**Keywords:** teicoplanin, therapeutic drug monitoring, hematological malignancies, model-informed precision dosing

## Abstract

**Background:** Teicoplanin is widely used for the empirical and targeted treatment of febrile neutropenia in patients with hematological malignancies. However, the pathophysiological alterations typical of this population may substantially affect drug exposure. The aim of this study was to develop and validate a population pharmacokinetic (PopPK) model of teicoplanin in adult hematological patients and to propose individualized dosing strategies. **Methods:** A retrospective, single-center study including 151 patients and 263 serum concentrations was conducted, with participants assigned to development (*n* = 100) and validation (*n* = 51) cohorts. Concentrations were quantified using a turbidimetric immunoassay, and the PopPK model was developed in NONMEM using FOCE-I. **Results:** Teicoplanin pharmacokinetics were described by a one-compartment model with first-order elimination. Ideal body weight, estimated glomerular filtration rate, and age were identified as significant predictors of clearance. Internal and external validation confirmed the robustness and predictive performance of the model. Monte Carlo simulations showed that conventional regimens (6 mg/kg every 12 h for three loading doses, followed by 6 mg/kg once-daily, or 600 mg every 12 h for three loading doses, followed by 600 mg once-daily) are insufficient to achieve therapeutic trough concentrations (≥15–20 mg/L) within the first 72 h, particularly in patients with preserved renal function or higher body weight. An intensified regimen consisting of five loading doses of 12 mg/kg every 12 h, followed by 12 mg/kg once-daily, enabled rapid attainment and maintenance of trough concentrations ≥ 20 mg/L in patients with lower to intermediate ideal body weight. **Conclusions:** These findings underscore the importance of intensified dosing strategies and covariate-guided individualization supported by therapeutic drug monitoring to achieve optimal teicoplanin exposure in this vulnerable patient group.

## 1. Introduction

Patients with hematological malignancies and febrile neutropenia are at high risk of infection, with Gram-negative bacteria representing the most frequently identified pathogens. However, infections caused by resistant Gram-positive cocci, such as methicillin-resistant *Staphylococcus aureus* (MRSA), also carry substantial clinical relevance, supporting the inclusion of Gram-positive coverage in empirical antibiotic regimens. In this context, teicoplanin is one of the most widely used therapeutic options in hematological patients because of its potent activity against resistant Gram-positive microorganisms and its favorable safety profile [1].

Hematological patients often exhibit several pathophysiological alterations including variability in renal function, hypoalbuminemia and changes in the apparent volume of distribution (Vd) that may significantly influence teicoplanin elimination and systemic exposure [2,3]. Augmented renal clearance (ARC) is particularly common in younger patients with good functional status who receive cytotoxic chemotherapy or intensive supportive care and may lead to markedly increased drug clearance (CL) and a consequent risk of subtherapeutic concentrations [4]. Hypoalbuminemia, which is prevalent in this population, may further affect the pharmacokinetics of highly protein-bound drugs such as teicoplanin. Because only the unbound drug is available for tissue distribution and elimination, reduced albumin levels promote greater tissue penetration and faster clearance from the central compartment, resulting in increases in both Vd and CL [5,6]. Altogether, these physiological alterations contribute to the considerable interindividual variability (IIV) observed in teicoplanin exposure.

Teicoplanin efficacy is primarily associated with the pharmacokinetic/pharmacodynamic (PK/PD) index defined by the ratio between the area under the concentration–time curve and the minimum inhibitory concentration (AUC/MIC) expressed as a time-based index. As estimating AUC requires multiple serial samples and is rarely feasible in clinical practice, trough concentrations (Cmin) are commonly used as a surrogate marker due to their strong correlation with AUC [6]. In MRSA infections, preclinical PK/PD models have shown that optimal bactericidal activity is achieved at a total AUC/MIC of 610, derived from isolates with an MIC of 0.5 mg/L, highlighting the MIC-dependent nature of this target [7]. Current recommendations suggest achieving Cmin > 15 mg/L in complicated infections and ≥20–30 mg/L in more severe conditions, such as infective endocarditis or osteoarticular infections [7,8,9,10]. For safety, maintaining Cmin values below 60 mg/L has been proposed, although supporting evidence remains limited [11].

Given the narrow therapeutic window and substantial variability in exposure, individualized dosing strategies are essential. Population pharmacokinetic (PopPK) models represent a key tool for characterizing IIV, identifying clinically important covariates, and guiding dose optimization through Bayesian forecasting. Despite the widespread use of teicoplanin in hematology settings, specific pharmacokinetic data in this population remain scarce, and the adequacy of conventional dosing regimens is not well established.

Population pharmacokinetic modeling and model-informed drug development approaches have been increasingly recognized by regulatory agencies as valuable tools to support dose optimization, particularly in special populations and clinical scenarios not fully addressed in pivotal clinical trials [12,13,14]. In this context, model-informed pharmacokinetic analyses provide a framework to evaluate alternative dosing regimens, identify patient subgroups at risk of under- or overexposure, and support rational dose individualization in complex clinical settings.

Therefore, the present study was designed with two objectives: (1) to develop and validate a PopPK model of teicoplanin in adult patients with hematological malignancies, characterizing the clinical and pathophysiological determinants of variability in drug exposure; and (2) to propose an optimized dosing strategy that enables early attainment of therapeutic concentrations and improves the effectiveness of empirical antibiotic therapy in this highly vulnerable population.

## 2. Materials and Methods

### 2.1. Study Design and Population

This was a retrospective, single-center, multidisciplinary study conducted in a tertiary-care hospital. The study received ethical approval from the Ethics Committee for Clinical Research of the hospital.

Hospitalized adult patients (≥18 years) with a diagnosis of malignant hematological neoplasms who received intravenous teicoplanin as part of the treatment for febrile neutropenia between February 2021 and December 2023 were eligible for inclusion. Patients with end-stage renal disease or receiving renal replacement therapy were excluded, as well as those with incomplete data or without valid teicoplanin concentration measurements. The first 100 patients enrolled were used for PopPK model development, and the 51 subsequent patients were allocated to the model validation cohort.

Patients initially received intravenous doses of 600 mg every 12 h, administered over 30 min [8]. Subsequent dosing was individualized according to therapeutic drug monitoring (TDM) results. The first serum teicoplanin concentration (STC) measurement was obtained 24 h after treatment initiation, and subsequent assessments were performed every 72–96 h, always immediately before dose administration (Cmin). Given the long half-life of teicoplanin, early measurements were not assumed to reflect steady-state conditions but were used to support dose individualization during both the loading and maintenance phases. The general target was to achieve Cmin > 15 mg/L, with a higher threshold (Cmin ≥ 20 mg/L) applied in suspected complicated infections, including persistent bacteremia, osteoarticular involvement, severe pneumonia, central nervous system infection, or high suspicion of uncontrolled MRSA infection.

The following variables were collected for each patient:

- Demographic variables: age, sex, body weight, height, ideal body weight (IBW; Devine formula), adjusted body weight (using a 0.4 correction factor for excess body weight), body mass index (BMI; weight/height^2^), and body surface area (BSA; Du Bois formula).
- Clinical variables: hematological diagnosis, teicoplanin dosing regimen, administered doses, and treatment duration.
- Biochemical variables: serum albumin, total protein, serum creatinine, C-reactive protein (CRP), hemoglobin, and platelet count. Renal function was estimated using the CKD-EPI (Chronic Kidney Disease Epidemiology Collaboration) equation to calculate the estimated glomerular filtration rate (eGFR).

### 2.2. Determination of Teicoplanin Concentrations

Serum teicoplanin concentrations were measured in the Clinical Pharmacokinetics Laboratory of the Pharmacy Department using a turbidimetric immunoassay (QMS^®^ Teicoplanin Assay, Thermo Fisher Scientific, Waltham, MA, USA) on an Abbott Architect ci4100 automated analyzer (Abbott Diagnostics, Abbott Park, IL, USA). Serum samples were obtained from whole blood collected in tubes without anticoagulant, centrifuged at 3000 rpm for 10 min, and stored at −20 °C until analysis.

The lower limit of quantification (LLOQ) of the assay was 3.0 mg/L, with a calibration range from 3.0 to 50 mg/L. The method demonstrated linearity within this interval, with deviations between −1.0% and 5.6%, well within the predefined acceptance criteria of ±15% according to CLSI EP6-A guidelines. Intra- and inter-day precision were below 10%, and accuracy was within ±15% of the nominal value. STC values below the LLOQ were excluded from the formal analysis [15].

### 2.3. Population Pharmacokinetic Model Development

A nonlinear mixed-effect modelling approach was used to develop the teicoplanin PopPK model, employing the First-Order Conditional Estimation method with interaction (FOCE-I) implemented in NONMEM^®^ version 7.5 (ICON Development Solutions, Ellicott City, MD, USA) [16,17]. Diagnostic evaluations and simulation procedures were performed in R version 4.5.0 (R Foundation for Statistical Computing, Vienna, Austria) [18].

A one-compartment model with first-order elimination was implemented to describe the pharmacokinetics of teicoplanin consistent with previously published population models in adult patients with MRSA infection [19]. The model was parameterized in terms of CL and Vd. The selection of the structural model was guided by the sampling design and data informativeness. As the dataset consisted exclusively of STC collected during the elimination phase, a parsimonious model structure was prioritized to ensure parameter identifiability and robustness. A two-compartment model was formally evaluated but did not result in a relevant improvement in model fit, as assessed by changes in the objective function value (ΔOFV ≈ 0.2) and showed poor precision of distribution parameters (relative standard errors (RSE) > 100%). Therefore, the one-compartment model was retained as the most robust structure for the available data. Interindividual variability in pharmacokinetic parameters was assumed to follow a log-normal distribution. Residual unexplained variability (RUV) was explored using proportional, additive, and combined (proportional + additive) error models. The magnitude of both IIV and RUV was expressed as a coefficient of variation (CV%). The most appropriate error structure was selected based on goodness-of-fit criteria, visual inspection of diagnostic plots, and changes in the OFV and parameter identifiability.

Once the structural and stochastic components of the base model had been established, all collected demographic, clinical, and biochemical variables were considered for the initial covariate analysis. The selection process included an initial screening based on physiological plausibility, visual inspection of the relationships between individual Bayesian estimates (η-values) of CL and Vd and potential covariates, and stepwise linear regression analyses to assess correlations between continuous covariates and η-values. For categorical covariates, differences were evaluated using ANOVA. Covariates were retained for further evaluation if they met the following criteria: physiological plausibility, adequate representation within the study population, statistical significance (*p* < 0.05), and a coefficient of determination (r^2^ > 0.10) with at least one pharmacokinetic parameter.

Selected covariates were subsequently evaluated individually using a stepwise covariate modelling (SCM) procedure implemented in NONMEM, applying forward inclusion (ΔOFV > 3.84; *p* < 0.05) and backward elimination (ΔOFV > 6.63; *p* < 0.01) criteria [20]. Time-dependent factors, such as fluctuations in renal function during treatment, were also explored through additional analyses in NONMEM. A summary of the univariate covariate analysis is provided in the Supplementary Materials (Table S1).

Selection of the final model was further supported by statistical stability, absence of bias in goodness-of-fit plots (GOF), and appropriate predictive performance assessed through standard population-modelling diagnostics [21].

### 2.4. Model Validation and Predictive Performance

The predictive performance of the final teicoplanin PopPK model was evaluated through both internal and external validation procedures. Internal validation was conducted using GOF plots, including observed versus population-predicted and individual-predicted STCs, as well as conditional weighted residuals (CWRES) versus population predictions and time after the first dose [22,23]. These diagnostic plots were visually inspected to identify potential trends, bias, or model misspecification. In addition, a non-parametric bootstrap analysis (1000 replicates) was performed to assess model robustness and parameter estimate precision, using random resampling with replacement of the original dataset in PsN version 4.9 [24]. Only runs that successfully converged were retained. Median values and 95% confidence intervals derived from the bootstrap distributions were used to evaluate the stability and precision of the model.

Internal validation also included a prediction-corrected visual predictive check (pcVPC), generated from 1000 simulations according to the methodological recommendations of Bergstrand et al. (2011) [25], to assess the ability of the model to replicate the observed variability over time.

External validation was performed using the patients recruited after completion of the model development cohort, forming an independent dataset not used for the initial model fitting. Predictive performance was evaluated using GOF plots and by quantifying accuracy and precision through the mean prediction error (*MPE*) and mean absolute prediction error (*MAPE*), respectively, calculated using the following expressions:

$$MPE\;=\;\frac{1}{n}\;\cdot \;\sum_{i\;=\;1}^{n}\frac{C_{pred,i}\;-\;C_{obs,i}\;}{C_{obs,i}}\cdot \;100\;MAPE\;=\;\frac{1}{n}\;\cdot \;\sum_{i\;=\;1}^{n}\left|\frac{C_{pred,i}\;-\;C_{obs,i}}{C_{obs,i}}\right|\cdot \;100$$

where

*C_pred,i_*: predicted concentration;

*C_obs,i_*: observed concentration;

*n*: total number of observations.

The MPE reflects the bias of the model predictions, whereas the MAPE quantifies predictive precision by expressing the mean absolute prediction error. Lower MAPE values indicate superior predictive performance. Both metrics were interpreted according to internationally accepted criteria for PopPK model validation [23].

### 2.5. Simulations and Probability of Target Attainment (PTA)

Monte Carlo simulations were performed using the final PopPK model to explore the influence of patient characteristics on teicoplanin exposure and to evaluate the probability of target attainment (PTA) for clinically relevant dosing regimens [26]. Each scenario was defined by representative combinations of demographic and clinical covariates within the range observed in the study population, and concentration–time profiles following multiple dosing were generated until steady-state was reached. For each dosing regimen, 1000 virtual individuals were simulated, sampling individual pharmacokinetic parameters according to the IIV estimated in the population model. The PTA was calculated as the proportion of simulated individuals achieving predefined STC thresholds (≥15 mg/L and ≥20 mg/L) at two clinically relevant time points: 72 h (after the loading phase) and at steady-state (trough concentration before the scheduled dose) [27].

In addition, to facilitate dose individualization, a dosing nomogram was generated based on simulation outputs to identify the minimum daily dose required to achieve therapeutic steady-state concentrations as a function of the relevant covariates identified in the model.

## 3. Results

### 3.1. Patient Characteristics

A total of 151 patients (65 women) with 263 STC measurements were included. Of these, the first 100 patients (168 concentrations) were assigned to the model development cohort, and 51 patients (95 concentrations) to the external validation cohort. All measured concentrations were above the LLOQ. Baseline clinical and demographic characteristics of the patients are presented in Table 1.

### 3.2. Population Pharmacokinetic Model

Teicoplanin pharmacokinetics were adequately described by a one-compartment model with first-order elimination parameterized in terms of CL and Vd with final parameter estimates summarized in Table 2.

In accordance with previous models in hematological patients and with physiological considerations, body-size metrics and renal function were assessed a priori as covariates on CL. Among the anthropometric descriptors evaluated (total body weight, adjusted body weight, IBW, BMI, and BSA), IBW was the descriptor that best explained interindividual variability in CL. Its inclusion as a power function normalized to the median population value (IBW/61)^3.2^, expressed in kilograms, resulted in a significant improvement in model fit and diagnostic performance and was therefore retained in the final model. Similarly, the eGFR, standardized to the population median value of 92.15 mL/min, was related to drug elimination through a power function (eGFR/92.15)^0.35^, consistent with the predominantly renal excretion of teicoplanin. In the covariate analysis, age also showed a significant effect on CL and was incorporated through a centered linear relationship: (1 + 0.01 × (AGE − 62)).

The combined inclusion of IBW, eGFR, and age reduced IIV in CL from 47.8% to 34.1%, and in Vd from 35.8% to 31.0%.

Final model equation for CL (functional form):

(1) $$CCL\;\left(\frac{L}{h}\right)=\;1.28\;\times \;\left(1+\;0.012\;\times \;\left(AGE\;-\;62\right)\right)\times \;\left({\left(\frac{eGFR}{92.15}\right)}^{0.35}\right)\times \;\left({\left(\frac{IBW}{61}\right)}^{3.2}\right)$$

where

IBW: ideal body weight.

For Men: IBW (kg) = 50.0 kg + 2.3 kg × ((height in cm − 152.4) − 2.54).

For Women: IBW (kg) = 45.5 kg + 2.3 kg × ((height in cm − 152.4) − 2.54).

AGE: age in years.

eGFR: estimated glomerular filtration rate (CKD-EPI formula) in mL/min.

The Vd was estimated at 92.1 L, a value consistent with those reported in the literature for teicoplanin in adult patients, reflecting its high plasma protein binding and extensive tissue distribution.

Residual unexplained variability (RUV) was modelled using an additive error structure, with an estimated value of 2.61 mg/L, representing the combined effect of analytical imprecision, incomplete model specification, and intraindividual variability. Proportional and combined residual error models were evaluated but did not result in an improvement in OFV or GOF diagnostics compared with the additive error model. Given the narrow range of observed concentrations and the predominance of trough samples, proportional error components were poorly identifiable and did not meaningfully contribute to model performance. Therefore, residual unexplained variability was described using a purely additive error model.

### 3.3. Model Evaluation

All pharmacokinetic parameters were estimated with adequate precision, as the RSE was ≤47% for all fixed-effect parameters. The η-shrinkage values were 23.9% for CL and 24.7% for Vd, indicating an acceptable degree of shrinkage that does not substantially compromise individual estimates or model diagnostics.

No significant differences were observed between the mean pharmacokinetic parameters obtained from the bootstrap analysis (*n* = 1000) and the final model estimates (Table 2). Moreover, all estimated parameters fell within the 95% confidence intervals (95% CI) derived from the bootstrap distributions, supporting the robustness of the final model.

Of the 1000 bootstrap runs, 6 estimates (0.6%) were discarded for being close to the boundaries of the parameter space, a proportion considered acceptable and without compromising the validity of the analysis. The remaining 994 successful runs confirmed the stability of the model. The bootstrap analysis also confirmed the precision of the covariate effect estimates, with confidence intervals that remained consistent across the resampled datasets (Table 2).

**Figure 1:**
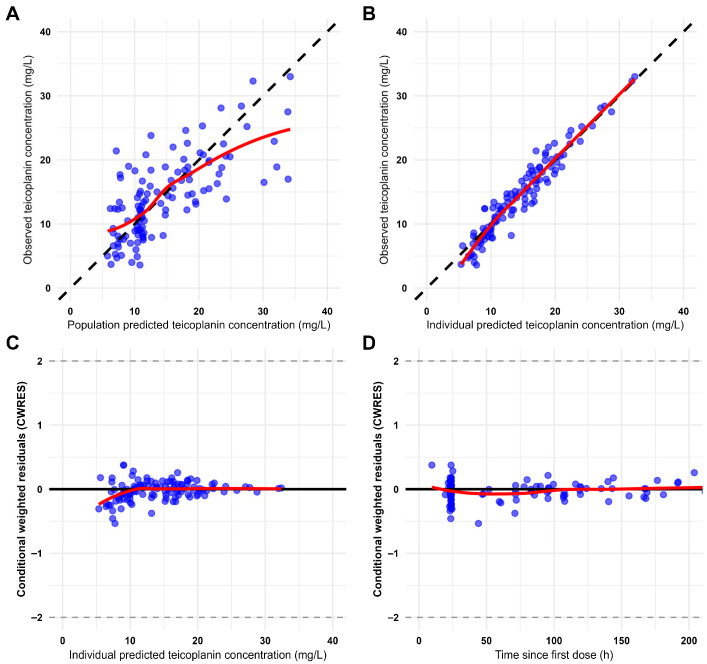
Goodness-of-fit plots for the final population model. Observed concentrations versus population-predicted concentrations (**A**); observed concentrations versus individual-predicted concentrations (**B**); conditional weighted residuals versus observed concentrations (**C**) and conditional weighted residuals versus time (**D**). Black solid line: line of identity; blue circles: teicoplanin concentrations; red solid line: locally weighted scatterplot smoothing (LOWESS).

The GOF plots for the final population model (Figure 1) showed no structural bias and demonstrated an adequate correlation between population- and individual-predicted concentrations and the observed values.

**Figure 2:**
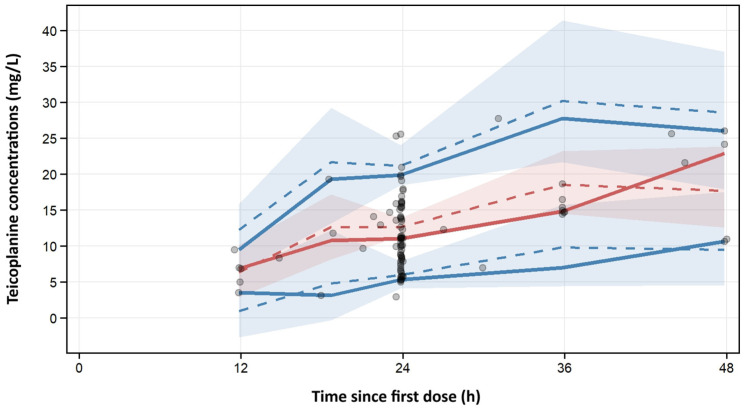
Prediction-corrected visual predictive check (pcVPC) for the concentration-time after-dose profiles of teicoplanin in hematological patients (internal evaluation). Gray open circles, teicoplanin observations (Obs); red solid line, median of the Obs; blue solid line, median of the predicted teicoplanin concentrations (Pred); red dashed lines, 5th and 95th percentiles of the Obs; blue dashed lines, 5th and 95th percentiles of the Pred (90% prediction interval, PI); blue-shaded area, 95% confidence interval (CI) for the 5th, 50th and 95th percentiles of the Pred; red-shaded area, 95% CI for the 5th, 50th and 95th percentiles of the Obs. The pcVPC was based on 1000 Monte Carlo simulations over a 48-h period following the first dose in 96 observations from the development dataset.

The pcVPC analysis, generated from 1000 Monte Carlo simulations over a 48-h period following the first dose showed that the model adequately reproduced both the central tendency and the variability of the teicoplanin concentration–time profile (Figure 2). Most observed concentrations fell within the 95% prediction intervals, with no evidence of systematic bias over time. The associated diagnostic metrics confirmed good predictive performance, with very low false-positive and false-negative rates (≤2.1%). Overall, these findings support the ability of the model to reliably describe and predict teicoplanin pharmacokinetics in patients with hematological malignancies.

### 3.4. Simulations and Probability of Target Attainment (PTA)

**Figure 3:**
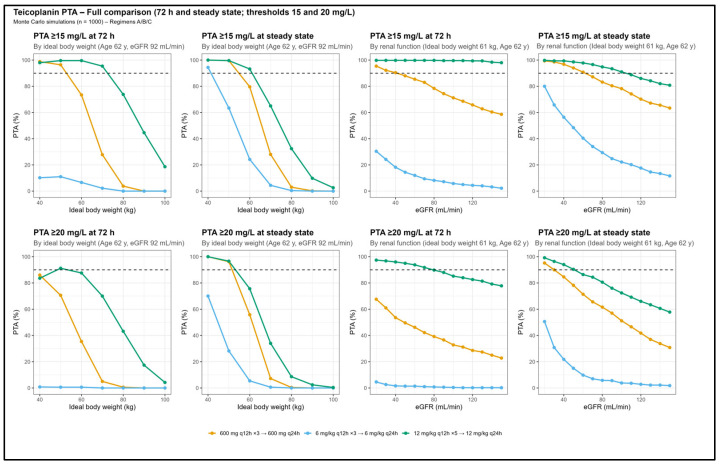
Probability of target attainment (PTA) for teicoplanin according to ideal body weight and renal function. Monte Carlo simulations based on the final population pharmacokinetic model assessing the probability of achieving trough concentrations ≥ 15 mg/L and ≥20 mg/L at 72 h (loading phase) and at steady state (maintenance phase). The upper panels depict PTA according to ideal body weight, and the lower panels according to estimated glomerular filtration rate (eGFR). Each line corresponds to a dosing regimen: Light blue: 6 mg/kg every 12 h × 3 → 6 mg/kg once-daily (summary of product characteristics); Yellow: 600 mg every 12 h × 3 → 600 mg once-daily (local protocol); Green: 12 mg/kg every 12 h × 5 → 12 mg/kg once-daily (proposed regimen). Horizontal dashed lines indicate a PTA threshold ≥ 90%, considered adequate.

Monte Carlo simulations showed that conventional teicoplanin regimens (three loading doses of 6 mg/kg every 12 h followed by once-daily maintenance dosing, or three loading doses of 600 mg every 12 h followed by a once-daily maintenance dose, reflecting commonly used weight-based and fixed-dose strategies in adult clinical practice) exhibited a suboptimal probability of achieving Cmin ≥ 15–20 mg/L during the first 72 h and at steady state, particularly in patients with higher body weight or preserved renal function (Figure 3). In contrast, the intensified regimen of five loading doses of 12 mg/kg every 12 h followed by a once-daily maintenance dose achieved a PTA greater than 90% both at 72 h and at steady state in patients with lower to intermediate IBW and reduced to moderate renal function, whereas PTA decreased in individuals with higher IBW or preserved renal function.

**Figure 4:**
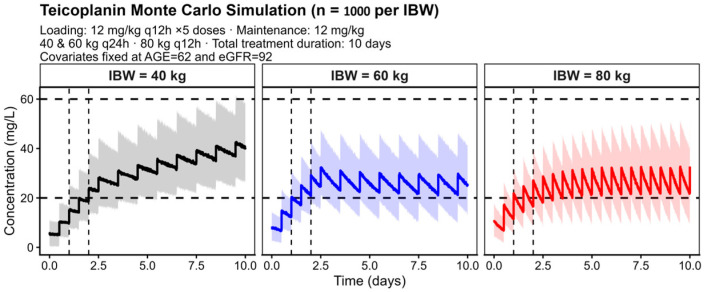
Simulated teicoplanin concentration–time profiles stratified by ideal body weight (IBW). Simulations were performed using the final population pharmacokinetic model for representative patients with IBW of 40, 60, and 80 kg following intravenous administration of teicoplanin. Solid lines represent the simulated mean concentrations, and shaded areas indicate the 90% prediction interval. Horizontal dashed lines indicate commonly used therapeutic (20 mg/L) and upper safety (60 mg/L) thresholds.

Stratified simulations by IBW (40, 60, and 80 kg) showed that the regimen of five doses of 12 mg/kg every 12 h followed by a once-daily maintenance dose enabled both attainment and maintenance of stable therapeutic teicoplanin concentrations within the first 72 h in individuals with an IBW ≤ 60 kg (Figure 4). In patients with an IBW ≥ 80 kg, once-daily dosing was insufficient to maintain stable therapeutic concentrations, with a tendency towards suboptimal levels during the maintenance phase and a need for higher daily doses. Therefore, an exploratory twice-daily (Q12 h) maintenance regimen was explored in this subgroup to assess whether increased dosing frequency could improve target attainment (Figure 4). As patients with IBW ≥ 80 kg were not represented in the model development dataset, these findings rely on model extrapolation and should be interpreted with caution.

**Figure 5:**
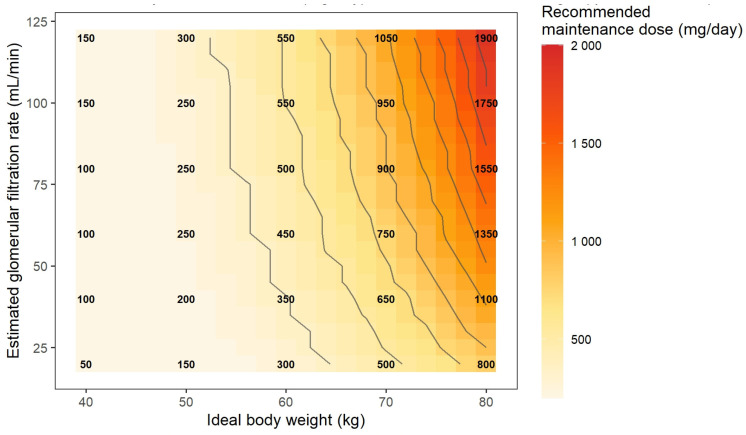
Individualized teicoplanin dosing nomogram for hematological patients. The chart illustrates the interaction between ideal body weight and estimated glomerular filtration rate (CKD-EPI equation) in determining the minimum daily maintenance dose (following five loading doses of 12 mg/kg every 12 h) required to achieve steady-state plasma concentrations within the therapeutic range (Cmin,ss ≥ 15 mg/L). Each intersection point represents a simulated combination of body weight and renal function, while the color scale indicates the recommended daily intravenous dose (mg/day).

Based on these simulations, a model-informed dosing nomogram was developed to estimate the minimum daily maintenance dose required to achieve steady-state Cmin ≥ 15 mg/L as a function of IBW and eGFR (Figure 5). The nomogram illustrates a proportional relationship between these covariates: patients with higher IBW and preserved renal function require larger daily doses (≥1500 mg/day), whereas those with lower body weight or reduced renal function achieve the therapeutic target with doses < 800 mg/day.

## 4. Discussion

Teicoplanin is widely used in patients with hematological malignancies and febrile neutropenia, both as empirical and targeted therapy against resistant Gram-positive bacteria, particularly MRSA and *Enterococcus* spp. Its favorable safety profile, once-daily administration, and activity against resistant pathogens support its frequent use in this clinical setting. However, the pathophysiological alterations characteristic of hematological patients, including variability in renal function, hypoalbuminemia, and changes in body composition, may substantially modify its pharmacokinetics, thus hindering the attainment of early therapeutic concentrations and increasing IIV in drug exposure [28].

In this study, we developed and validated a PopPK model of teicoplanin specifically in adult patients with hematological malignancies, a population in which pharmacokinetic data remain limited. Teicoplanin pharmacokinetics were adequately described by a one-compartment model with first-order elimination. Although teicoplanin has often been described using multi-compartment models [4,6], the identification of distribution parameters requires sampling during the early distribution phase. In the present study, the use of Cmin only limited the ability to reliably estimate such parameters. Consistently, a two-compartment model did not provide a meaningful improvement in model fit and showed poor parameter precision, supporting the use of a parsimonious one-compartment structure. Renal function, age, and IBW were identified as significant covariates explaining interindividual variability in clearance.

Renal function, characterized by eGFR, showed a direct association with CL, consistent with the predominantly renal elimination of teicoplanin and with findings from heterogeneous or critically ill populations [29,30]. IBW, compared with other anthropometric descriptors such as total or adjusted body weight, emerged as the most relevant predictor of CL in this cohort. This finding may reflect the influence of altered body composition frequently observed in patients with hematological malignancies, including cachexia, fluid overload, and malnutrition, where total body weight may overestimate the metabolically active mass contributing to drug clearance [28]. Although age remained in the model as a covariate with a positive coefficient on CL, this does not necessarily reflect a true physiological increase in renal elimination. More plausibly, it represents an apparent increase in total CL driven by lower albumin concentrations and altered body composition in older or cachectic patients. Increased protein catabolism associated with systemic inflammation, malignancy, and anticancer treatments contribute significantly to hypoalbuminemia, particularly in elderly individuals [5,6]. Given the high plasma protein binding of teicoplanin, reduced albumin concentrations increase the unbound fraction of the drug and, consequently, its apparent CL, even in the absence of changes in renal function [5]. This mechanism has also been described for other highly protein-bound antibiotics administered in malnourished or chronically inflamed populations [1]. The inclusion of these three covariates reduced IIV in CL from 47.7% to 34.1%, markedly improving model fit and predictive performance relative to previous models such as that of Zhang et al. [15], which reported greater parameter dispersion and lower population homogeneity.

Unlike previous studies, our model focuses exclusively on patients with hematological malignancies, a population characterized by high PK variability and limited representation in the literature. In this regard, the models by Sako et al. [4] and Byrne et al. [6] identified renal function (serum creatinine or creatinine clearance), total body weight, and serum albumin as significant predictors of CL, underlining the importance of renal elimination and plasma protein binding in teicoplanin pharmacokinetics. In contrast, in our model IBW emerged as the most appropriate anthropometric descriptor, providing a better representation of metabolically active body size and avoiding overestimation of CL in overweight patients or those with oedema, conditions common in this population [31].

Albumin has been identified as a relevant covariate for CL in other models [6,19,32]. In our cohort, this variable did not reach statistical significance, likely due to consistently low albumin levels, its partial collinearity with age, and the use of total rather than unbound STC, which may limit the ability to detect its true contribution.

External validation confirmed the predictive performance of the model, with acceptable accuracy and precision and no evidence of systematic bias between predicted and observed concentrations. GOF plots and statistical indicators (MPE and MAPE) showed satisfactory agreement between model predictions and external data, supporting its robustness and clinical applicability. Collectively, these findings validate the model as a useful tool to support teicoplanin dose individualization in hematological patients, ensuring adequate predictive performance beyond the development dataset.

From a clinical perspective, the long elimination half-life of teicoplanin delays the achievement of steady state, potentially compromising adequate exposure during the first days of treatment [3], a particularly relevant limitation in hematological patients [33]. Our findings corroborate this issue, as conventional regimens showed insufficient probability of achieving early therapeutic concentrations in patients with high IBW or preserved renal function (Figure 3).

The intensified regimen of five loading doses of 12 mg/kg every 12 h highlights the need for more aggressive or individualized dosing strategies to ensure adequate early exposure, especially in patients with physiological characteristics favoring increased CL. This dosing strategy was selected based on previously published population pharmacokinetic studies and clinical experience supporting the use of intensified teicoplanin loading regimens to rapidly achieve therapeutic concentrations [6,27], and is consistent with approved dosing recommendations for severe infections such as infective endocarditis and bone and joint infections. Likewise, IBW-stratified simulations (Figure 4) indicate that patients with higher body mass may require adjustments to the maintenance dose or dosing frequency to maintain stable therapeutic concentrations.

From a safety perspective, the use of an intensified loading regimen warrants careful consideration, particularly regarding the risk of nephrotoxicity. In the present model-based simulations, the proposed loading strategy (12 mg/kg every 12 h for five doses) was not predicted to result in STC exceeding levels that have been commonly associated with an increased risk of nephrotoxicity (approximately 60 mg/L) across the simulated scenarios [11]. However, during the maintenance phase, a subset of patients with lower IBW (≤40 kg) was predicted to reach concentrations above this threshold at steady state. This finding underscores the importance of early and individualized TDM to guide maintenance dose adjustment and prevent excessive exposure. Therefore, the proposed dosing strategy should be interpreted as a model-informed framework to support individualized dosing, to be implemented in conjunction with routine TDM and close monitoring of renal function, rather than as a fixed dosing recommendation.

The dosing nomogram developed (Figure 5) provides a practical tool in support of dosing individualization based on key variables such as IBW and eGFR, facilitating the implementation of a model-informed precision dosing (MIPD) approach in the hematology setting. Incorporating this strategy may contribute to optimizing early exposure and therapeutic response, particularly in scenarios characterized by high PK variability and clinical severity.

The modeling and simulation framework applied in this study is consistent with current regulatory recommendations supporting the use of PopPK and model-informed approaches for dose optimization, particularly in special populations. Recent regulatory experience highlights the increasing role of pharmacokinetic modeling in informing dosing strategies beyond pivotal trials, including in oncology drug development and regulatory assessments [34,35].

This study has several strengths. First, it constitutes one of the few PopPK analyses of teicoplanin conducted exclusively in patients with hematological malignancies, a population with substantial pathophysiological variability and limited representation in prior literature. Second, the model was externally validated with an independent dataset, supporting its robustness and predictive ability in real-world clinical scenarios. Additionally, the use of real-world clinical data and the integration of clinically relevant covariates (IBW, renal function, and age) enhance its applicability for dose individualization. Finally, the development of model-based graphical tools, such as PTA simulations and the dosing nomogram, facilitates translation of the model into clinical practice.

Nonetheless, the study has limitations. Its single-center design may restrict the generalizability of the results to other populations or centers with different demographic characteristics or treatment practices. Moreover, total STC were used without distinguishing the unbound fraction. Teicoplanin is known to be highly protein-bound in human plasma, with a reported unbound fraction of approximately 5–10%, and the use of total concentrations may underestimate the influence of hypoalbuminemia on drug disposition. However, this approach is consistent with current clinical practice, as therapeutic targets and routine TDM are based on total concentrations. In addition, the number of observations in patients with extreme body weights or severely reduced eGFR was limited; thus, model predictions in these subgroups should be interpreted with caution and always complemented by individualized TDM. Finally, correlations between serum concentrations and clinical or microbiological outcomes were not assessed. As a result, the proposed dosing recommendations are based on pharmacokinetic targets and model-based simulations rather than on direct evidence of clinical efficacy or safety. Prospective studies validating the model in external cohorts and assessing its impact on clinical efficacy and safety would therefore be desirable.

## 5. Conclusions

The PopPK model developed in this study adequately characterizes teicoplanin pharmacokinetics in adult patients with hematological malignancies and provides a useful tool to support individualized dosing optimization. Our findings highlight the need for higher loading and maintenance doses than those recommended in the summary of product characteristics to achieve early therapeutic concentrations in this vulnerable population, which is highly susceptible to invasive infections. Moreover, incorporating simple clinical covariates such as IBW and/or renal function allows for more precise adjustment of maintenance dosing.

Finally, combining these strategies with therapeutic drug monitoring (TDM) is recommended to ensure optimal exposure, maximize clinical efficacy, and minimize toxicity risk, thereby promoting the rational and safe use of teicoplanin in hematological patients.

## Figures and Tables

**Table 1:** Demographic and Clinical Data of Patients in the Development and Validation Cohorts.

Characteristics	Development Cohort	Validation Cohort
Total number of patients	100	51
Sex [female], %	40	49.01
Age, median [range], years	62 [18–85]	58 [23–87]
Weight, median [range], kg	68 [41–127]	70 [43–130]
Ideal body weight, median [range], kg	61.4 [46.25–77]	62.75 [47–80]
Biochemical parameters, median [range] Serum albumin, g/dL	3.3 [2.4–4.3]	3.0 [1.4–4.6]
Total protein, g/dL	5.4 [3.7–8.7]	5.6 [2.6–7.3]
Serum creatinine, mg/dL	0.71 [0.30–2.11]	0.62 [0.12–3.12]
Estimated glomerular filtration rate *, mL/min/1.73 m^2^	92.15 [26–141]	98.77 [15–145]
C-reactive protein, mg/dL	7.82 [7.82–43.54]	8.22 [0.13–47.84]
Hemoglobin, g/dL	9.2 [6.9–13.3]	9.3 [7.0–14]
Platelet count, /µL	36,500 [3000–565,000]	31,000 [2900–385,000]
Diagnosis		
Non-Hodgkin’s lymphoma, %	20	31.37
Acute myeloblastic leukemia, %	20	21.57
Multiple myeloma, %	20	21.57
Acute lymphoblastic leukemia, %	9	9.8
Other hematologic malignancies, %	31	15.69
Dosage, median [range], mg/kg	9.1 [3–14.6]	8.7 [4.6–18.6]
Loading phase, mg/kg	8.8 [6.3–14.6]	8.6 [6.2–14.0]
Maintenance phase, mg/kg	9.2 [3–14.5]	8.8 [4.6–18.6]
Plasma samples (n)	168	95
Loading phase, %	60%	54%
Maintenance phase, %	40%	46%
Mean concentration (SD), mg/L	14.37 (7.20)	14.08 (6.09)

* Estimated glomerular filtration rate calculated using the CKD-EPI equation (mL/min).

**Table 2:** Teicoplanin population pharmacokinetic parameters.

Parameters	Final Model Estimate	RSE (%)	Shrinkage	Bootstrap (*n* = 1000)	
				Mean	95% CI
CLpop (L/h)	1.28	7	23.9	1.26	1.11–1.42
eGFR-CL	0.35	47		0.33	0.06–0.67
IBW-CL	3.20	21		3.25	2.13–4.78
AGE-CL	0.01	16		0.01	0.01–0.02
Vpop (L)	92.10	5	24.7	92.36	85.25–100.50
IIVCL (CV, %)	34.10	23		31.60	23.2–39.80
IIVV (CV, %)	31.0	26		30.10	21.0–39.7
RUVadi (mg/L)	2.61	21		2.59	1.93–3.03

AGE, age in years; AGE-CL, magnitude of the effect of AGE on CL; CI, confidence interval; CLpop, clearance of the typical subject; eGFR, estimated glomerular filtration rate (CKD-EPI formula) in mL/min.; eGFR-CL, magnitude of the effect of eGFR on CL; IBW-CL, ideal body weight in kg; IBW-CL, magnitude of the effect of IBW on CL; IIVCL, interindividual variability on clearance; IIVV, interindividual variability on volume of distribution; RSE, relative standard error; RUVadi, additive error of residual variability; Vpop, volume of distribution of the typical subject.

## Data Availability

The original contributions presented in this study are included in the article. Further inquiries can be directed to the corresponding author.

## Supplementary Materials

The following supporting information can be downloaded at: https://www.mdpi.com/article/10.3390/pharmaceutics18010100/s1, Table S1. Results of the univariate covariate analysis evaluating the effects of anthropometric, demographic, and clinical covariates on teicoplanin pharmacokinetic parameters.

## Abbreviations

The following abbreviations are used in this manuscript:

ARC	Augmented renal clearance
AUC/MIC	Ratio between the area under the concentration–time curve and the minimum inhibitory concentration
BMI	Body mass index
BSA	Body surface area
CL	Drug clearance
Cmin	Trough concentration (immediately before the next dose)
CWRES	Conditional weighted residuals
eGFR	Estimated glomerular filtration rate
IBW	Ideal body weight
MAPE	Mean absolute prediction error
MPE	Mean prediction error
MRSA	Methicillin-resistant *Staphylococcus aureus*
OFV	Objective function value
pcVPC	Prediction-corrected visual predictive check
PopPK	Population pharmacokinetic
PTA	Probability of target attainment
RSE	Relative standard error
RUV	Residual unexplained variability
STC	Serum teicoplanin concentration
TDM	Therapeutic drug monitoring
Vd	Volume of distribution
