# Supplementary material for: Population Pharmacokinetics and Model-Informed Dose Optimization of Teicoplanin in Adults with Hematological Malignancies
Source: Pharmaceutics. 2026 Jan 12;18(1):100. doi: 10.3390/pharmaceutics18010100 (PMC12844755; doi:10.3390/pharmaceutics18010100)
Supplement: Supplementary file 1 [file pharmaceutics-18-00100-s001.zip › pharmaceutics-4048474-supplementary.pdf]

Supplementary Table S1. Results of the univariate covariate analysis evaluating the effects of anthropometric, demographic, and clinical covariates on teicoplanin pharmacokinetic parameters.

PK parameter	Covariate	Best functional form	ΔOFV	Passed inclusion criterion	Retained in final model
CL	Ideal body weight (IBW)	Power	15.59	Yes	**Yes**
CL	Adjusted body weight	Power	14.37	Yes	No
CL	Total body weight	Power	11.15	Yes	No
CL	Body mass index (BMI)	Power	3.75	No	No
CL	Age	Linear	1.85	No	**Yes***
CL	Renal function (eGFR)	Power	2.43	No	**Yes***
CL	Albumin	Linear	3.20	No	No
CL	Hemoglobin	Power	2.46	No	No
CL	Platelet count	Power	5.03	Yes	No
CL	C-reactive protein	Linear	3.83	No	No
CL	Sex	Categorical	16.28	Yes	No
CL	Diagnosis	Categorical	12.89	No	No
CL	Body Surface area (BSA)	Linear	15.50	Yes	No
Vd	Total body weight	Power	19.99	Yes	No
Vd	Adjusted body weight	Power	18.77	Yes	No
Vd	Ideal body weight (IBW)	Power	10.90	Yes	No
Vd	Body mass index (BMI)	Power	11.57	Yes	No
Vd	Age	Power	12.00	Yes	No
Vd	Sex	Categorical	10.99	Yes	No
Vd	Diagnosis	Categorical	28.89	Yes	No
Vd	Body Surface area (BSA)	Power	22.71	Yes	No

Univariate covariate screening was performed using a stepwise covariate modeling (SCM) approach.

For continuous covariates, linear and power relationships were evaluated; the table reports the functional form yielding the largest decrease in objective function value (ΔOFV) for each covariate–parameter relationship.

Covariates were considered statistically significant if ΔOFV exceeded 3.84 for 1 degree of freedom. Categorical covariates with multiple degrees of freedom (e.g., diagnosis) were evaluated using the appropriate likelihood ratio test threshold.

Supplementary Table S1. Results of the univariate covariate analysis evaluating the effects of anthropometric, demographic, and clinical covariates on teicoplanin pharmacokinetic parameters.

PK parameter	Covariate	Best functional form	ΔOFV	Passed inclusion criterion	Retained in final model

Covariate effects on the volume of distribution (Vd) were explored but were not retained in the final model due to limited information on distribution parameters in the trough-only sampling design and insufficient parameter precision.

*Although age and renal function did not meet the predefined inclusion criterion in the initial univariate screening, both covariates became statistically significant after adjustment for anthropometric descriptors and were therefore retained in the final multivariate model.
